# Rho family GTPase-dependent immunity in plants and animals

**DOI:** 10.3389/fpls.2014.00522

**Published:** 2014-10-14

**Authors:** Yoji Kawano, Takako Kaneko-Kawano, Ko Shimamoto

**Affiliations:** ^1^Laboratory of Plant Molecular Genetics, Nara Institute of Science and TechnologyIkoma, Japan; ^2^College of Pharmaceutical Sciences, Ritsumeikan UniversityKusatsu, Japan

**Keywords:** Rac/Rop, small GTPase, plant immunity, PAMPs-triggered immunity, effector-triggered immunity

## Abstract

In plants, sophisticated forms of immune systems have developed to cope with a variety of pathogens. Accumulating evidence indicates that Rac (also known as Rop), a member of the Rho family of small GTPases, is a key regulator of immunity in plants and animals. Like other small GTPases, Rac/Rop GTPases function as a molecular switch downstream of immune receptors by cycling between GDP-bound inactive and GTP-bound active forms in cells. Rac/Rop GTPases trigger various immune responses, thereby resulting in enhanced disease resistance to pathogens. In this review, we highlight recent studies that have contributed to our current understanding of the Rac/Rop family GTPases and the upstream and downstream proteins involved in plant immunity. We also compare the features of effector-triggered immunity between plants and animals, and discuss the *in vivo* monitoring of Rac/Rop activation.

## INTRODUCTION

Recent studies on plant-pathogen interactions have revealed that plants have developed a two-branched system of immunity to prevent the invasion of pathogens. The perception of pathogen/microbe-associated molecular patterns (PAMPs/MAMPs) by host pattern recognition receptors (PRRs) is important for the initiation step of innate immunity (Jones and Dangl, 2006; Dodds and Rathjen, 2010; Monaghan and Zipfel, 2012). In plants, host perception of PAMPs activates rapid defense responses such as the production of reactive oxygen species (ROS), induction of defense-related genes, and MAPK activation, designated PAMP-triggered immunity (PTI). Most PRRs are subdivided into three categories that include receptor-like kinases (RLKs), receptor-like proteins (RLPs), and receptor-like cytoplasmic kinases (RLCK; Monaghan and Zipfel, 2012). RLKs consist of an extracellular domain, a transmembrane domain, and an intracellular kinase domain, whereas RLPs lack the intracellular kinase domain and RLCKs only possess a cytoplasmic kinase domain. Structural studies show that RLKs perceive signals through their extracellular domains and transmit signals through their intracellular signaling molecules using their kinase domains. The *Arabidopsis* and rice genomes encode more than 600 and 1100 RLKs/RLCKs, respectively, that participate in various cellular signaling processes and developmental events (Shiu et al., 2004). Chitin, found in pathogenic and non-pathogenic fungi, is one of the best-characterized PAMPs (Gust et al., 2012). Two lysine motif (LysM)-containing PRRs, OsCEBiP, and OsCERK1, play a vital role in chitin signaling (Kaku et al., 2006; Shimizu et al., 2010). OsCEBiP is an RLP that lacks an intracellular kinase domain and directly binds chitin, whereas OsCERK1 is an RLK and does not directly bind chitin. These two immune proteins form a receptor complex that transduces the chitin signals to downstream components (Kaku et al., 2006; Shimizu et al., 2010; Shinya et al., 2012).

To promote pathogen virulence, pathogen effectors target plant proteins and, thus, perturb host cell physiology and immunity. As a result, a second line of plant defense counterattacks the pathogens. This defense system is termed effector-triggered immunity (ETI; Jones and Dangl, 2006; Dodds and Rathjen, 2010). Compared to PTI, the ETI response is often more robust and faster. Disease resistance (R) proteins act as intracellular receptors for the direct or indirect recognition of specific pathogen effectors [also called avirulence (Avr) proteins]. ETI triggered by R proteins results in strong host responses accompanied by cell death. Most R genes encode members of the nucleotide-binding (NB) domain and leucine-rich repeat (LRR) domain (NLR, also called NB-LRR) family that often display a tripartite domain architecture and are subdivided according to their N-terminal domains into coiled-coil (CC-NLR) and Toll/Interleukin-1 Receptor homology (TIR-NLR) subclasses. Although sharing broad structural similarities, many NLR proteins also show unique structural variations that are important for their function and subcellular localization (Lukasik and Takken, 2009).

The Rho family of GTPases belongs to the Ras superfamily of small GTPases. The Rho family in animals is further divided into 3 subfamilies: the Rho, Rac, and Cdc42 proteins. In contrast, the Rho family in plants converges into a single subfamily that is distinct from all major subfamilies of animal Rho-GTPases (Li et al., 1998). The plant Rho subfamily is most closely related to the animal Rac subfamily (about 65% identity at the amino acid level). Thus, these plant GTPases are called Rac-like (Rac family) or Rop-like (Rho-related GTPases of plants) proteins (Kawasaki et al., 1999; Winge et al., 2000). The Rac/Rop family is one of the most important regulators of signal transduction in plants, participating in pathways that influence growth and development and the adaptation of plants to various environmental situations (Berken, 2006). Evidence is accumulating that the Rac/Rop family plays a critical role in plant immunity (Kawano et al., 2010b; Kawano and Shimamoto, 2013). Among the Rac/Rop family of small GTPases in plants, we will highlight two small GTPases in the Rac/Rop family, rice *Oryza sativa* Rac1 (OsRac1) and barley *Hordeum vulgare* RacB (HvRacB), in this review.

## Rac/Rop FAMILY OF SMALL GTPases IN PLANTS

**FIGURE 1:**
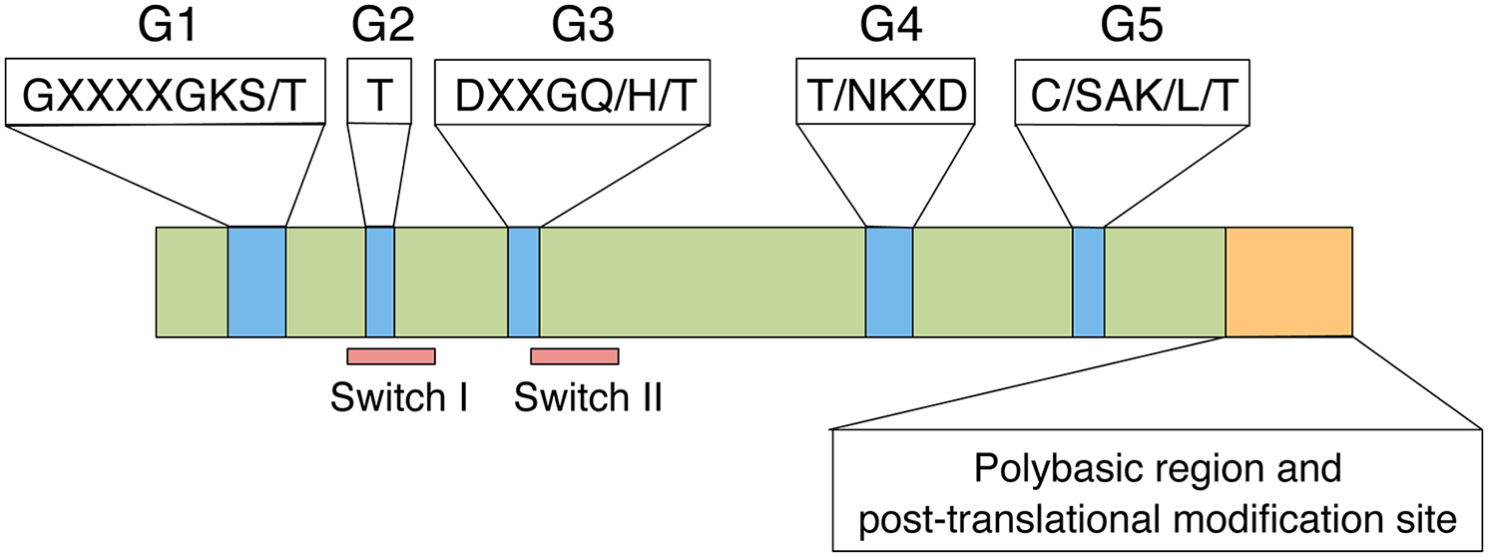
**Functional domains of the Rac/Rop family.** The Rac/Rop family shares a set of five conserved G boxes (Blue), a polybasic region and a post-translational modification site (Orange).

Proteins in the Rac/Rop GTPase family contain five highly conserved G-boxes (G1–G5; **Figure 1**; Paduch et al., 2001; Wennerberg et al., 2005). G1, G3, G4, and G5 play critical roles in binding to GTP/GDP and hydrolysing GTP to GDP. The G2 box is known to be the effector domain that is essential for binding to downstream effector proteins. The C-terminal polybasic region and post-translational modification site play important roles in subcellular localization and small GTPase function. Rac/Rop GTPases can be divided into two types based on their C-terminal motifs (Winge et al., 2000). Type I Rac/Rop GTPases possess a conserved CaaL (a: aliphatic amino acid) motif, whereas type II proteins lack this motif but retain a cysteine-containing element for membrane anchoring. All type-I Rac/Rop family members are putatively prenylated; the type-II proteins are palmitoylated but not prenylated (Lavy et al., 2002).

There are seven *Rac/Rop* family genes in rice (Miki et al., 2005), 6 genes in barley (Schultheiss et al., 2003), and 11 genes in *Arabidopsis* (Winge et al., 2000). All seven members of the Rac/Rop family in rice are expressed in seedlings, leaf sheaths, stems, and roots, but expression of *OsRac2*, *6*, and *7* is much lower than that in leaf blades (Chen et al., 2010b). The expression level of *OsRac7* is also low in panicles, immature seeds, and cultured cells. These different tissue specificities suggest distinct roles for different Rac/Rop small GTPases in the various signaling pathways in rice. OsRac5–7 are type I Rac/Rop proteins that have a conserved CaaL motif at the C-terminus, and OsRac1-4 are type II Rac/Rop proteins that possess a truncated but functional post-translational modification motif (Chen et al., 2010b). In general, members of the Rac/Rop family are localized mainly at the plasma membrane, but some signals are detected in the cytoplasm and the nucleus (Chen et al., 2010b). Members of the rice type I Rac/Rop family are more often localized in the nuclei and the cytoplasm than the type II proteins. Constitutively active (CA) forms of OsRac/Rops tend to show plasma membrane localization more often than their dominant negative (DN) forms. OsRac3 and OsRac4 have the highest percentage of plasma membrane localization among the rice Rac/Rop GTPases.

## REGULATORS OF Rac/Rop GTPases

**FIGURE 2:**
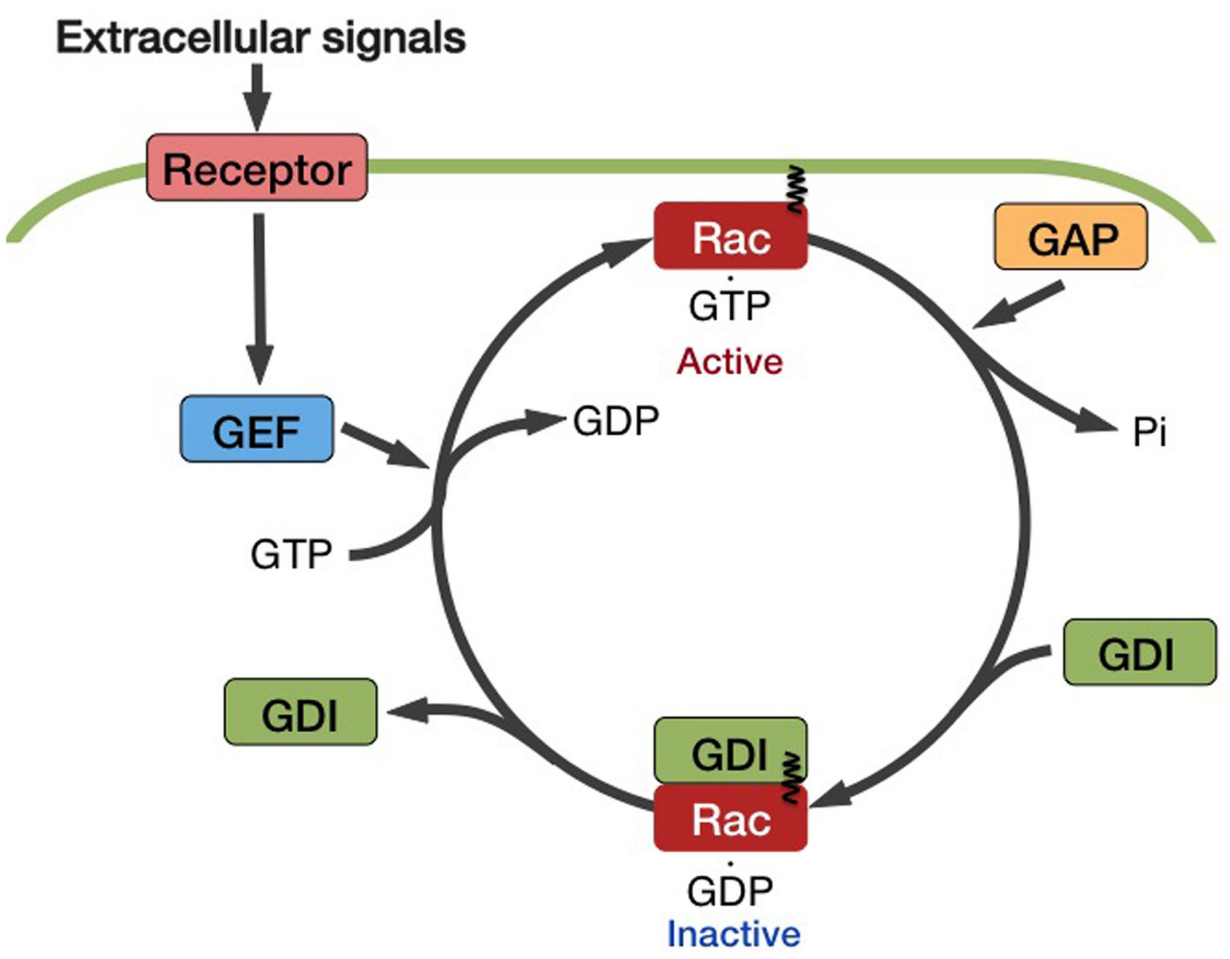
**Mode of activation of the Rac/Rop family GTPases.** The ratio of these two forms of Rac/Rop is dependent of the activity of regulating factors. GTPase-activating proteins (GAPs) act as negative regulators by accelerating the intrinsic GTPase activity of Rac/Rop and reconverting it to the inactive GDP.Rac/Rop. Guanine nucleotide dissociation inhibitors (GDIs) inhibit the exchange of GDP for GTP. Guanine nucleotide exchange factors (GEFs) facilitate the release of GDP from Rac/Rop, thereby promoting the binding of GTP.

The ratio between the GDP-bound inactive and GTP-bound active forms of Rac/Rop depends on the activity of regulating proteins (**Figure 2**). GTPase-activating protein (GAP) works as a negative regulator by promoting the intrinsic GTPase activity of Rac/Rop and reconverting it to the inactive GDP ⋅ Rac/Rop. Guanine nucleotide dissociation inhibitor (GDI) inhibits the exchange of GDP for GTP. Guanine nucleotide exchange factor (GEF) enhances the release of GDP from Rac/Rop, thereby promoting the binding of GTP. GTP⋅Rac/Rop interacts with downstream effectors and then triggers various cellular responses.

More than 30 RacGEFs have been described in animals; most of them share conserved Dbl homology (DH) and pleckstrin homology (PH) domains (Bos et al., 2007). Notably, only two DH–PH RacGEFs with significant similarity to human SWAP70 have been recently found in plants (Shinohara et al., 2002; Yamaguchi et al., 2012). SWAP70 contains both DH and PH domains, but their arrangement is the reverse of that in typical DH-PH-type Rho GEFs, wherein the DH domain is flanked by a C-terminal PH domain. In addition, plants possess a unique family of RacGEFs whose members specifically activate Rac/Rop GTPases *in vitro* (Berken et al., 2005). RacGEFs are characterized by a highly conserved catalytic domain called a plant-specific Rop nucleotide exchanger (PRONE). PRONE was found to promote nucleotide dissociation from Rac/Rop with catalytic properties comparable to DH-PH GEFs. Based on the three-dimensional structure of PRONE GEF, catalysis follows a push-and-pull mechanism affecting the switch regions of small GTPases (Thomas et al., 2007).

## Rac/Rop IN PTI

Constitutively active form (CA)-OsRac1 causes hypersensitive response (HR)-like responses and greatly reduces disease lesions induced by virulent races of the rice blast fungus (Kawasaki et al., 1999; Ono et al., 2001). CA-OsRac1 also causes resistance against virulent races of bacterial blight, enhances production of phytoalexins, and alters expression of defense-related genes. These results indicate that OsRac1 acts as a positive regulator of PTI in rice. To further elucidate the roles of all seven Rac/Rop family proteins in rice immunity, we made knockdown plants of each *O*s*Rac* family gene and performed the infection assays using a virulent race of rice blast fungus (Chen et al., 2010b). *OsRac4* and *OsRac5* RNAi plants have shorter lesions caused by a virulent race of rice blast fungus, suggesting that OsRac4 and OsRac5 are negative regulators of PTI. There are no obvious effects in *OsRac3*, *OsRac6*, and *OsRac7* RNAi plants on the lesions induced by a virulent race of rice blast fungus. OsRac6 plays a modest role in defense; however, based on an overexpression study, *OsRac6*, also known as *OsRacB*, was proposed to be a negative regulator in defense (Jung et al., 2006). The existence of positive and negative roles for Rac/Rop GTPases in rice innate immunity also indicates the complexity of Rac/Rop functions in disease resistance.

## Rac/Rop AND IMMUNE RECEPTORS IN PTI

Studies in other research areas provide clues for how PRRs activate Rac/Rop family GTPases in plant immunity. The Rac/Rop family is implicated in signaling downstream of RLK CLAVATA1, a receptor regulating the balance between cell differentiation and cell division in aerial meristems (Trotochaud et al., 1999). An unidentified Rac/Rop GTPase was found in the immunoprecipitate of the 450 kDa active CLAVATA1 complex. Moreover, McCormick and colleagues found that PRONE-type AtRopGEF associates with pollen-specific RLKs, LePRK1, and LePRK2 (Kaothien et al., 2005). They characterized AtPRK2a, an *Arabidopsis* homolog of LePRK2, and verified the physical interaction between AtPRK2a and the *Arabidopsis* PRONE-type AtRopGEF12, implying that RopGEF activity is regulated by RLKs. Phosphorylation of the C-terminus of AtRopGEF12 appears to be important for regulating GEF activity (Zhang and McCormick, 2007). A phospho-mimicking mutation at a highly conserved serine residue in the C-terminus of AtRopGEF12 results in the loss of the C-terminal autoinhibition.

**FIGURE 3:**
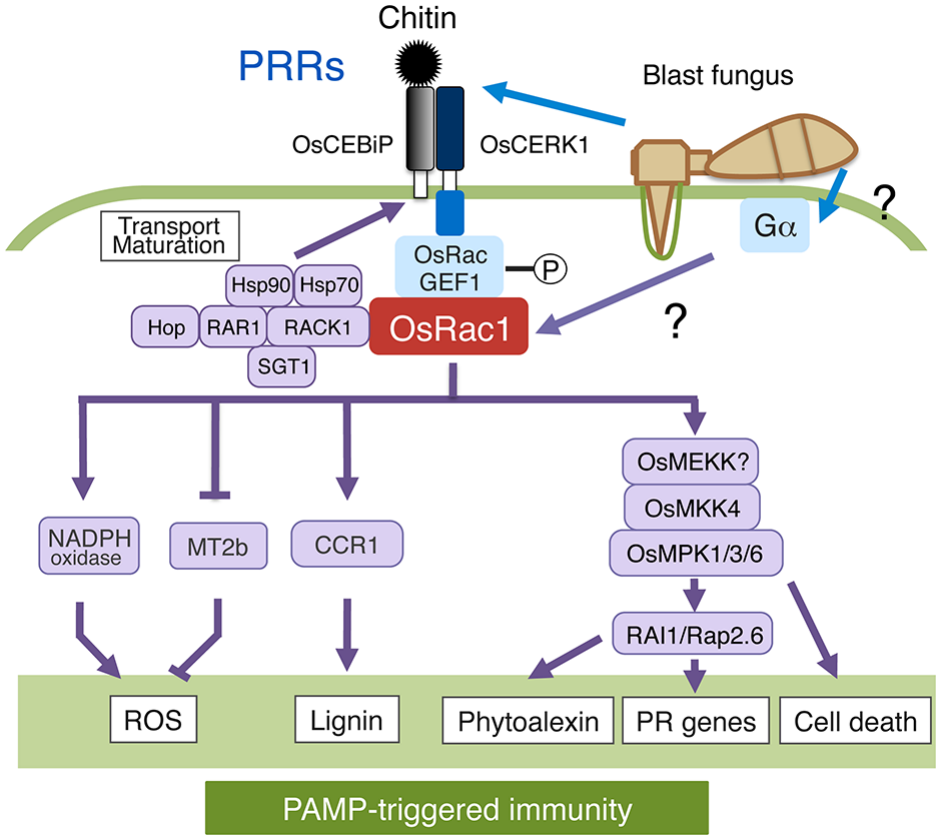
**Roles of OsRac1 in PAMP-triggered immunity (PTI).** OsCERK1-dependent phosphorylation of OsRacGEF1 leads to the activation of OsRac1. OsRac1 activates OsMPK1/3/6 through OsMEKK4; activated OsMPK3/6 activates the transcription factor RAI1 as well as Rap2.6. The chaperone and co-chaperone complex of Hop/Sti1-Hsp90 facilitates the maturation and transport of OsCERK1. OsRac1 plays a dual role as an activator of NADPH oxidase and a suppressor of reactive oxygen species (ROS) scavenging. Lignin: cell wall, Phytoalexin: antibacterial substance, PR genes: Pathogenesis-related genes.

*Oryza sativa* Rac1 has been shown to participate in PTI induced by elicitors derived from fungal pathogens such as chitin and sphingolipids (Ono et al., 2001; Suharsono et al., 2002). How the OsCEBiP-OsCERK1 chitin-receptor complex transmits extracellular chitin signals to downstream components was unclear until recently. An OsCEBiP/OsCERK1-OsRacGEF1-OsRac1 module participates in the immunoresponse caused by chitin, and chitin activates OsRac1 within 3 min of chitin treatment (**Figure 3**; Akamatsu et al., 2013). OsRac1 interacts with a PRONE-type GEF called OsRacGEF1 that is directly phosphorylated at Ser549 after chitin treatment by OsCERK1. This phosphorylation leads to the activation of OsRacGEF1, resulting in the activation of OsRac1. Knockdown of *OsRacGEF1* compromises the expression of defense-related genes and attenuates disease resistance to virulent races of rice blast fungus. Overall, these results support the hypothesis that OsRacGEF1 is a direct substrate of OsCERK1 chitin receptor and that OsRacGEF1-phosphorylated by OsCERK1 leads to OsRac1 activation. A GEF protein, SWAP70, also interacts with OsRac1 and the DH-domain exhibited GEF activity toward OsRac1 *in vitro*, indicating that plants possess a functional DH domain (Yamaguchi et al., 2012). In addition, OsSWAP70 regulates chitin-induced ROS production and defense gene expression in rice. Thus, it is likely that SWAP70 functions as a GEF for OsRac1 in rice.

AtROP6 orchestrates developmental and disease resistance signaling (Poraty-Gavra et al., 2013). *AtROP6* expression is induced by the plant hormone auxin and is detected in the root meristem, lateral root initials, and leaf hydathodes. The expression of *DN-AtRop6* induces small, multiple inflorescence stems, twisted leaves, deformed leaf epidermis pavement cells, and differentially organized cytoskeletons. The expression of *DN-AtRop6* leads to major changes in gene expression for proteins participating in constitutive salicylic acid (SA)-mediated defense responses. Accordingly, the free and total SA levels in *DN-AtRop6* without an infection resemble those of wild-type plants inoculated with a virulent powdery mildew pathogen. The constitutive SA responses in *DN-AtRop6* are suppressed in mutants defective in SA signaling [non-expressors of PR gene1 (npr1)] or biosynthesis [SA induction deficient2 (sid2)]. However, the *DN-AtRop6 npr1* and *DN-AtRop6 sid2* double mutants retain the aberrant developmental phenotypes, implying that *AtRop6*’s function in development is not related to the constitutive SA response. *DN-AtRop6* plants exhibit the developmental phenotype of enhanced pre-invasive defense responses to a host-adapted virulent powdery mildew fungus, *Golovinomyces orontii*, but are impaired in pre-invasive defenses upon inoculation with a non-adapted powdery mildew, *Blumeria graminis* f. sp. hordei (*Bgh*). The host-adapted powdery mildew, *G. orontii*, has a reduced reproductive fitness on *DN-AtRop6* plants, a phenotype that is retained in mutants defective in SA biosynthesis or signaling. These results indicate that both the morphological aberrations and the enhanced disease resistance effect to host-adapted *G. orontii* in *DN-AtRop6* are independent of SA-dependent defense signaling.

## Rac/Rop AND NADPH OXIDASE IN PTI

In plants, ROS can strengthen host cell walls via glycoprotein cross-linking or cause lipid peroxidation and membrane damage. However, ROS are one of the most important second messengers in plant defense (Torres and Dangl, 2005). Additional regulatory functions for ROS in defense occur in conjunction with other plant signaling molecules, particularly SA and nitric oxide. Cotton (*Gossypium hirsutum*) Rac13 triggers the production of ROS, which may serve as a signal for secondary wall formation in cotton (Potikha et al., 1999). The CA mutant of barley HvRac1 promotes ROS accumulation in infected leaves (Pathuri et al., 2008); however, this effect is observed only in cells where a fungal attempt to penetrate failed, i.e., where neither a fungal haustorium initial nor an elongated secondary fungal hyphae developed. In contrast, there are no detectable effects on ROS production from the expression of two other *HvRacs*, *CA-HvRacB*, and *CA-HvRac3.* Rop guanosine triphosphatase activating protein 4 (RopGAP4) in *Arabidopsis* is involved in the generation of ROS required for responses to oxygen deprivation (Baxter-Burrell et al., 2002).

*Oryza sativa* Rac1 is a regulator of ROS production and cell death in rice (Kawasaki et al., 1999). CA-OsRac1 enhances PAMPs–induced ROS production and resistance to pathogens in rice (**Figure 3**; Kawasaki et al., 1999; Ono et al., 2001). The direct interaction between OsRac1 and the N-terminal region of NADPH oxidase [also called Respiratory burst oxidase homologs (Rboh)], including the two EF-hand motifs which is the most common calcium-binding motif, is required for the activation of Rboh by OsRac1 (Wong et al., 2007). The cytosolic Ca^2+^ concentration may modulate NADPH oxidase activity by regulating the direct interaction between OsRac1 and OsRboh. Structural analyses further support the hypothesis of a direct interaction between OsRac1 and RbohB (Oda et al., 2010; Kosami et al., 2014). The OsRac1 binding interface in OsRbohB is located in the flanking region of the coiled-coil region at the N-terminus. The structure of this binding region is not similar to those previously identified as Rac binding motifs in animals. Thus, OsRac1 binds to OsRbohB in a manner distinct from known interactions between Rac and its target proteins. The expression of *Metallothionein2b* (*MT2b*), a ROS scavenging gene, is synergistically down-regulated by OsRac1 and rice blast-derived elicitors (Wong et al., 2004). Collectively, OsRac1 might play a dual role as an inducer of ROS production and a suppressor of ROS scavenging.

## Rac/Rop AND MAPK SIGNALING IN PTI

MAPK signaling in all eukaryotes is organized in three-tiered modules comprising a MAPK kinase kinase (MAPKKK/MEKK), a dual-specificity MAPKK (MKK), and a MAPK (MPK), within which phosphorylation signals are transduced linearly from the MAPKKK to the MAPK (Rodriguez et al., 2010; Samajova et al., 2013). The CA MKK NtMEK2 activates two MPKs, NtSIPK, and NtWIPK, followed by an induced HR and defense gene expression (Yang et al., 2001). A complete MPK cascade involving MEKK1-MKK4/MKK5-MPK3/MPK6 has been previously reported in *Arabidopsis* (Asai et al., 2002). OsMPK1 protein levels are strongly reduced in *OsRac1*-knockdown cells and in the trimeric G-protein α subunit mutant *d1*, and sphingolipid elicitor-induced OsMPK1 activation is greatly reduced in both mutant cells (**Figure 3**; Lieberherr et al., 2005). These results suggest that these two GTP-binding proteins contribute to the stability of OsMPK1 protein and, possibly, for its activation as well. In animals, Ras-like GTPases are involved in the upstream signaling for MAPK cascade activation. These Ras-MAPK or G-protein-MAPK cascades occur in response to various stimuli, such as hormones or environmental stresses. Gα functions upstream of OsRac1 in the sphingolipid elicitor signaling pathway, leading to the induction of ROS production and defense gene expression (Suharsono et al., 2002). Therefore, a MAPK cascade may be located downstream of these two G-proteins along with other pathways. In fact, our immunoprecipitation assay showed that OsMPK1 and OsRac1 form the same protein complex, indicating that OsRac1 activates OsMPK1 in a manner similar to the Ras-MAPK pathway in animals. The mechanism of how Gα activates OsRac1 in plants remains to be studied.

The basic helix–loop–helix transcription factor *Rac Immunity1* (*RAI1*) is up-regulated 15–30 min after chitin treatment (**Figure 3**; Kim et al., 2012). Moreover, the expression of *CA-OsRac1* up-regulates *RAI1* expression in rice suspension cells. Accordingly, *RAI1* T-DNA activation-tagged lines show enhanced resistance to a virulent race of blast fungus, implying that RAI1 is a positive regulator of plant immunity and is involved in the OsRac1-dependent chitin pathway. A microarray analysis of cells transformed with an inducible *RAI1* construct showed increased gene expression of *PAL1* and the transcription factor *OsWRKY19* after induction, suggesting that these genes are regulated by RAI1. Chitin elicitor activates *Oryza sativa* MAPK kinase 4 (OsMKK4) as well as two MAPKs, OsMPK3, and OsMPK6 (Kishi-Kaboshi et al., 2010). OsMKK4-dependent phosphorylation of OsMPK3 and OsMPK6 appears to be essential for the chitin elicitor-induced biosynthesis of diterpenoid phytoalexins that act as toxins to restrict *Magnaporthe oryzae* infection. OsMAPK3 and OsRac1 form the same complex as previously reported for OsMPK1 (Lieberherr et al., 2005; Kim et al., 2012). The expression levels of the two downstream genes, *PAL1* and *OsWRKY19*, are increased by overexpression of OsMPK6 and/or OsMPK3 together with the active form of OsMKK4. Moreover, RAI1, a transcription factor, is directly phosphorylated by OsMPK3/6 in an active form of OsMKK4-dependent manner *in vitro*. Taken together, our results indicate that RAI1 is regulated by OsRac1 through an OsMPK3/6 cascade. Similarly, a different type of transcription factor OsRap2.6 may be controlled by OsRac1 through an OsMPK3/6 pathway (Wamaitha et al., 2012).

## Rac/Rop AND DOWNSTREAM PROTEINS IN PTI

*Oryza sativa* Rac1 appears to form a “defensome network” consisting of various proteins that collectively regulate rice immunity (**Figure 3**; Kawano et al., 2010b; Kawano and Shimamoto, 2013). This network might include chitin receptor OsCERK1 as well as OsRacGEF1, the heat shock protein 90 (Hsp90), Hsp70, co-chaperone Hop/Sti1, the scaffold protein RACK1, the lignin biosynthesis enzyme *O. sativa* Cinnamoyl-CoA reductase 1 (OsCCR1), OsMPK3, OsMPK6, and RAI1 (Lieberherr et al., 2005; Thao et al., 2007; Nakashima et al., 2008; Chen et al., 2010a; Kim et al., 2012; Akamatsu et al., 2013). Hop/Sti1a and Hsp90 directly interact with OsCERK1 (Chen et al., 2010a). Co-chaperone Hop/Sti1 is transported from the endoplasmic reticulum (ER) to the plasma membrane, and the Hop/Sti1-Hsp90 chaperone complex contributes to the maturation and intracellular transport of the OsCERK1 complex (Chen et al., 2010a; Akamatsu et al., 2013). In fact, the knockdown of *Hop/Sti1* suppresses chitin-triggered pathogenesis-related gene expression and disease resistance to virulent races of rice blast fungus. The transport of the OsCERK1 complex is mediated by the small GTPase Sar1 that regulates ER-to-Golgi trafficking because overexpression of CA-Sar1 compromised the transport of OsCERK1 from the ER to the plasma membrane (Chen et al., 2010a). These results suggest that the Hop/Sti1-HSP90 chaperone complex plays an important role in the maturation and transport of PRRs and may function to link PRRs and Rac/Rop GTPases.

RACK1 associates with many signaling proteins in animals and acts as a scaffolding protein in a number of signaling pathways (McCahill et al., 2002). Although RAR1, Hsp90, and Hsp70 are present in the OsRac1 complex, none of them appear to interact directly with OsRac1 (**Figure 3**; Thao et al., 2007). OsRac1 appears to form a complex with these chaperones and co-chaperones through RACK1 because RACK1 directly interacts with OsRac1 as well as with SGT1, RAR1, and Hsp90 (Nakashima et al., 2008). The interaction of these three (co)-chaperones in rice seems to occur mainly in PTI (Thao et al., 2007; Wang et al., 2008). Accordingly, treatment with geldanamycin, an Hsp90 inhibitor, compromises OsRac1-HSP90 complex formation (Thao et al., 2007). RACK1 also plays a key role in the production of ROS and PTI (Nakashima et al., 2008). RACK1 is involved in hormone signaling and development in plants (Chen et al., 2006; Nakashima et al., 2008). OsRac1 positively regulates RACK1 at both the transcriptional and posttranscriptional levels. RACK1 transcription is also induced by chitin, a fungal elicitor, and by various plant hormones including abscisic acid, jasmonate, and auxin. RACK1 interacts with the N-terminus of NADPH oxidase, together with RAR1 and SGT1. Based on these results, two functions for RACK1 in rice innate immunity can be envisaged. One hypothesis is that RACK1 is a component of the defensome network consisting of OsRac1, RAR1, SGT1, Hsp90, and Hsp70 and functions as a scaffolding protein for this immune complex. Another possible function is that RACK1 is a component of the NADPH oxidase complex together with OsRac1 and regulates ROS production at an early stage in immune responses.

Lignin, a major component of secondary cell walls, is a heterogeneous tridimensional phenolic polymer resulting from the oxidative polymerization of monolignols (Boerjan et al., 2003). OsCCR1, an enzyme involved in lignin biosynthesis, is a target protein of OsRac1 (**Figure 3**; Kawasaki et al., 2006). Lignin is an important factor in plant defense responses because it forms an undegradable mechanical barrier to most pathogens. Sphingolipid treatment induces the expression of OsCCR1. OsRac1 binds OsCCR1 in a GTP-dependent manner, and the interaction of OsCCR1 with OsRac1 leads to the enzymatic activation of OsCCR1 *in vitro*. Suspension cells expressing CA-OsRac1 accumulate lignin through enhanced OsCCR1 activity and increased ROS production. Thus, OsRac1 likely controls lignin synthesis through regulation of both NADPH oxidase and OsCCR1 activities during defense responses.

## Rac/Rop IN ETI

**FIGURE 4:**
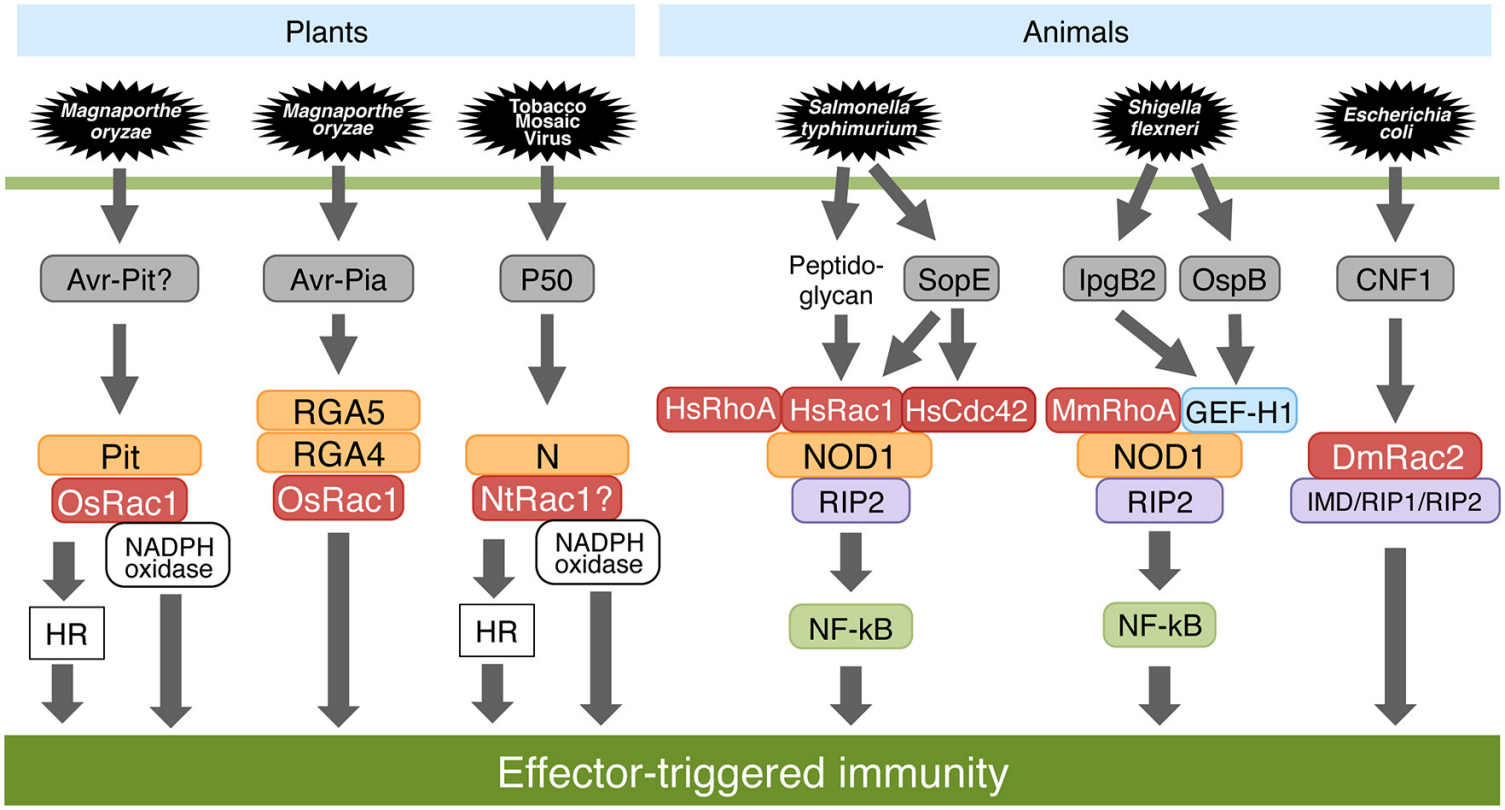
**Effector-triggered immunity (ETI) in plants and animals.** OsRac1 contributes to Pit- and Pia-mediated ROS production as well as the hypersensitive response (HR) in rice and is required for disease resistance to avirulent races of blast fungus. Tobacco NtRac1 also acts as a downstream molecule of N resistance protein. In human and mouse, an NLR protein, NOD1, monitors the activation state of the Rho family GTPases that are targeted by virulence effectors caused by pathogenic microbes. In flies, an *Escherichia coli*-derived effector molecule CNF1 modifies *Dm*Rac2 to trigger an immune response.

*Oryza sativa* Rac1 is involved in PTI as well as ETI including *Pi-a* and *Pit*-mediated disease resistance (**Figure 4**; Ono et al., 2001; Chen et al., 2010b; Kawano et al., 2010a,b; Kawano and Shimamoto, 2013). *Pi-a* and *Pit* are the resistance genes to rice blast fungus. OsRac1 interacts directly with the NB-ARC domain of Pit at the plasma membrane (Kawano et al., 2010a). OsRac1 is activated by the active form of Pit at the plasma membrane and thereby induces ROS production as well as the HR. Recently, to decipher the mechanisms involved in the localization of Pit, we searched for consensus sequences in Pit that are associated with membrane localization and found a pair of potential palmitoylation sites in the N-terminal coiled-coil region (Kawano et al., 2014). Although wild-type Pit is localized predominantly to the plasma membrane, this membrane localization was compromised in a Pit mutant in which a pair of cysteine residues that are potential palmitoylation sites were substituted with Alanines, indicating that palmitoylation is required for the plasma membrane localization of Pit. This palmitoylation-deficient Pit mutant has a significantly lower affinity for OsRac1 on the plasma membrane, resulting in failed Pit-mediated cell death, ROS production, and disease resistance to rice blast fungus. These results indicate that palmitoylation-dependent membrane localization of Pit is required for the interaction with and the activation of OsRac1, and that OsRac1 activation by Pit is vital for Pit-mediated disease resistance to rice blast fungus. Terauchi and colleagues cloned *Pia*, resistance genes, and found that the *Pia* locus contains two NLR-type *R* genes, *RGA4*, and *RGA5*, that are located next to each other in the genome and are oriented in opposite directions (Okuyama et al., 2011). RGA4 and RGA5 are a pair of R proteins and act together to trigger disease resistance against pathogens. Thus, the deletion of either of these R proteins fails to induce *Pia*-mediated resistance. RGA4 and RGA5 form a hetero-complex and interact through their coiled-coil domains. Recently, the different roles of RGA4 and RGA5 were resolved (Cesari et al., 2013; Césari et al., 2014). RGA5 directly interacts with and recognizes of the *M. oryzae* effector Avr-Pia as well as Avr1-CO39 and acts as a sensor for effector proteins. Interestingly, RGA4 and RGA5 have opposing functions: RGA4 constitutively induces immune responses, whereas RGA5 suppresses RGA4-induced immune responses. These findings raise interesting questions about how these two R proteins activate OsRac1. Moreover, overexpression of *DN-OsRac1* in tobacco leaves suppresses the synchronous production of HR and ROS triggered by *N* as well as *Pto* resistance genes (Moeder et al., 2005). Overall, it is likely that OsRac1 generally functions downstream of several R proteins.

Many previous studies using inhibitors and agonists of heterotrimeric G-proteins in several plant species have suggested that G-proteins are involved in defense signaling. The *d1* mutant showed that Gα is involved in disease resistance (Suharsono et al., 2002). *d1* mutants exhibit a highly reduced HR to infection by an avirulent race of rice blast fungus and enhanced hyphal extension, indicating that Gα is involved in *R*-gene-mediated disease resistance in rice. Activation of *PBZ1* expression, a pathogenesis-related gene, in *d1* with rice blast fungus is delayed for 24 h relative to the wild type (WT). *Gα* expression is induced by an avirulent race of rice blast, and the expression of *CA-OsRac1* in *d1* mutants restores sphingolipid elicitor-dependent *PBZ1* expression and disease resistance to an avirulent rice blast fungus. These results imply that the heterotrimeric G-protein functions upstream of OsRac1 in the early steps of signaling. Gα is also involved in *PBZ1* expression-induced by the plant activator probenazole (Iwata et al., 2003). The expression of CA-OsRac1 also induces the expression of *PBZ1*. Thus, OsRac1 is probably activated by probenazole downstream of Gα.

Phosphatidic acid (PA) is involved in numerous stress responses of plants. Intracellular PA levels increase under various biotic and abiotic stress conditions, including pathogen infection (Young et al., 1996; van der Luit et al., 2000); however, the physiological roles of PA in the stress response remain largely unclear. Treatment with PA induces cell death and elevates the levels of ROS in the leaves and single cells of *Arabidopsis* (Park et al., 2004). *Arabidopsis* leaves expressing a *CA-AtRop*2 develop earlier cell death and higher levels of ROS production than WT, whereas cell death in those expressing a *DN-AtRop2* is later and ROS production is lower. However, in the absence of exogenous PA, spontaneous cell death or ROS induction does not occur in *CA-AtRop2* plants, indicating that the activation of AtRop2 is required for ROS production but is not sufficient to induce the ROS generation pathway. These results suggest that PA regulates additional pathways required for active AtRop2-dependent ROS production. Therefore, PA may be an important regulator of AtRop2-mediated ROS generation and the cell death process during various stress and defense responses in *Arabidopsis*.

At present, we do not know the mechanism by which OsRac1, a single Rho family GTPase, has highly diverse functions. Differences in tissue distribution *in planta* or subcellular distribution in cells might lead to the functional diversity observed in OsRac1, but further studies are necessary to resolve this issue.

## Rac/Rop AND THE CYTOSKELETON IN SUSCEPTIBILITY TO DISEASE

**FIGURE 5:**
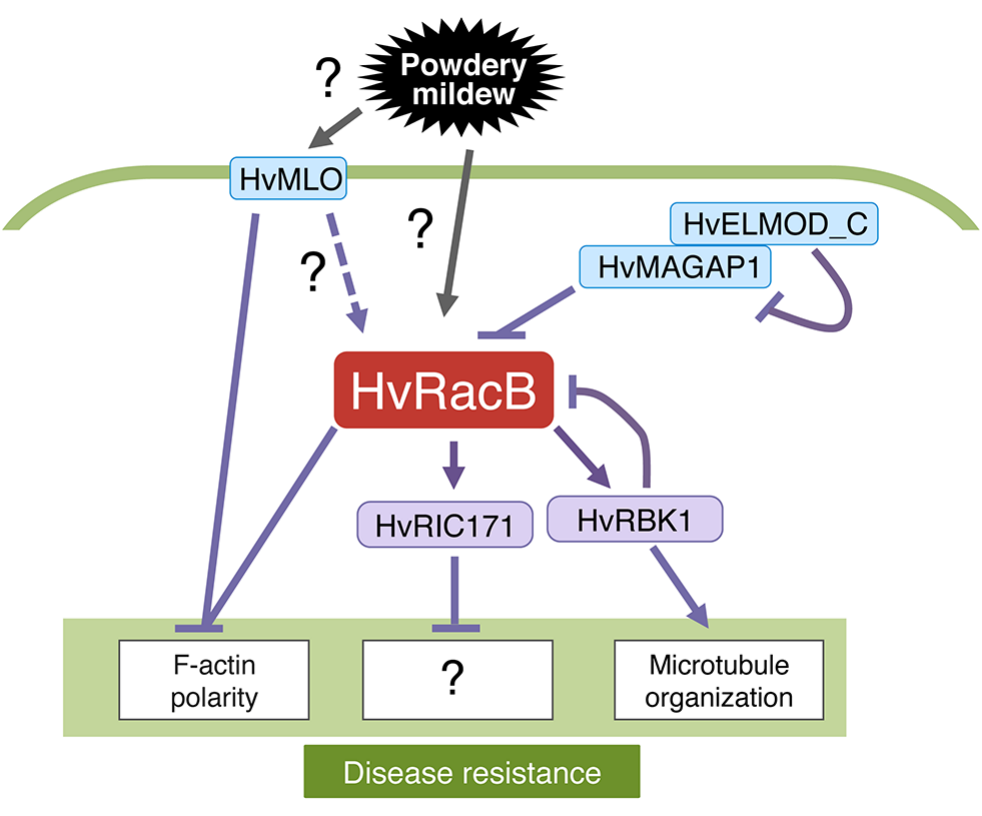
**Roles of *Hordeum vulgare* RacB (HvRacB) in plant immunity.** HvRacB is a major factor in MILDEW LOCUS O (MLO)-dependent disease susceptibility that negatively controls cytoskeleton-mediated defense responses at the cell wall. The polarized pattern of the cytoskeleton toward sites of fungal attack is closely related to successful defense of fungal invasion. HvRacB and MLO are host proteins involved in the modulation of the actin cytoskeleton at the interface between barley and its adapted powdery mildew fungus. HvRacB and its downstream effector, HvRIC171, act as susceptibility factors in the barley–powdery mildew interaction. Activity of HvRacB is controlled by microtubule-associated MICROTUBULE-ASSOCIATED ROP-GTPASE ACTIVATING PROTEIN1 (MAGAP1) whose function is again modulated by HvELMOD_C. The kinase HvRBK1 can be activated by HvRacB but functions in basal resistance to powdery mildew by influencing microtubule organization. This process might involve a negative feedback mechanism on HvRacB; however, it remains unclear, how MLO or HvRacB are activated.

Barley HvRacB contributes to the plant’s susceptibility to barley powdery mildew (Schultheiss et al., 2002). The function of HvRacB is related to that of the major susceptibility factor MILDEW LOCUS O (MLO; **Figure 5**) and *ROR1*, another locus that is required for recessive *mlo*-specified resistance (Schultheiss et al., 2002, 2003). The expression of CA-HvRacB causes enhanced susceptibility to penetration and haustorium formation by the barley powdery mildew fungus *Bgh* and causes depolarized growth of root hairs (Schultheiss et al., 2005; Pathuri et al., 2008). By contrast, silencing of *HvRacB* by RNAi limits fungal success in haustorium formation and causes a failure of root hair outgrowth (Hoefle et al., 2011). The biotrophic powdery mildew fungus *Bgh* penetrates susceptible barley (*Hordeum vulgare* L.) by invading epidermal cells that remain intact during fungal development. The actin cytoskeleton is differentially reorganized in susceptible and resistant plants challenged by *Bgh*. Actin filaments are highly polarized toward the sites of attempted penetration of *Bgh* in resistant plants, whereas a more subtle reorganization takes place around fungal haustoria in susceptible plants (Opalski et al., 2005). Polarized distribution of the actin cytoskeleton toward sites of fungal attack is closely related to the successful prevention of fungal invasion. Moreover, overexpression of *CA-HvRacB* partly inhibits the polarized distribution of F-actin toward sites of *Bgh* invasion, whereas knockdown of *HvRacB* enhances actin focusing. Overall, HvRacB and MLO are host proteins involved in the modulation of the actin cytoskeleton at the interface between the host plant and *Bgh*. CA-HvRacB interacts with HvRIC171, a barley Cdc42/Rac interactive binding domain (CRIB)-motif protein. CA-HvRacB and fungal attack promote recruitment of HvRIC171 to the cell periphery or sites of fungal entry, respectively, (Schultheiss et al., 2008). The overexpression of *HvRIC171*, similar to that of *CA-HvRACB*, renders the plant more susceptible to invasion by *Bgh*, whereas, expression of a 46-amino-acid HvRIC171-CRIB peptide, which is sufficient to interact with CA-HvRacB, has a DN effect and decreases susceptibility to *Bgh*. Taken together, these results suggest HvRacB and its downstream effector HvRIC171 act as susceptibility factors in the barley–powdery mildew interaction.

A barley MICROTUBULE-ASSOCIATED ROP-GTPASE ACTIVATING PROTEIN1 (MAGAP1) also interacts with HvRacB (**Figure 5**; Hoefle et al., 2011). MAGAP1 is localized along cortical microtubules and is recruited by activated HvRacB to the cell periphery. During fungal attack, MAGAP1-labeled microtubules form a polarized network at sites of successful defense. By contrast, microtubules loosen at the invasion sites where the fungus succeeds. A MAGAP1 mutant lacking GAP activity demonstrated that MAGAP1 is a limiting factor for susceptibility to penetration by *Bgh*. Moreover, MAGAP1 regulates the polarized distribution of cortical microtubules toward sites of infections. This finding supports the hypothesis that HvRacB and MAGAP1 act antagonistically in cytoskeleton organization during fungal entry (Hoefle et al., 2011). Engulfment and Motility (ELMO) proteins participate in the regulation of small GTPase activity in eukaryotic organisms. The barley ELMO-Domain Containing Protein (HvELMOD_C) is partially associated with microtubule-associated MAGAP1 (Hoefle and Huckelhoven, 2014). The expression of HvELMOD_C compromises the resistance-inducing effect of HvMAGAP1 to *Bgh* when simultaneously expressed with HvMAGAP1. Thus, it is likely that HvELMOD_C works as a new modulator of Rac/Rop signaling in barley. Furthermore, CA-HvRacB interacts with a ROP-binding protein kinase (HvRBK1; Huesmann et al., 2012). The kinase activity of HvRBK1 is enhanced by the addition of CA-HvRacB *in vitro*. *HvRBK1* RNAi enhanced the penetration of barley epidermal cells by *Bgh* and lowered the stability of cortical microtubules. Thus, HvRBK1 might function in basal resistance to powdery mildew by influencing microtubule organization or by a negative feedback on the susceptibility factor HvRacB. Further studies are necessary to elucidate how HvRacB orchestrates the two cytoskeletons, actin, and microtubules, during powdery mildew infection.

## Rho PROTEINS AND ETI IN ANIMALS

Recent studies have revealed that the involvement of the Rho family of proteins in NLR-dependent ETI signaling is conserved between plants and animals (**Figure 4**; Stuart and Boyer, 2013). In fact, co-immunoprecipitation assays with Rac1 show that *Homo sapiens* Rac1 (HsRac1) associates with NLR proteins NB oligomerization domain-containing protein 1 (NOD1) as well as NOD2, and OsRac1 directly interacts with the NLR protein Pit in rice (Legrand-Poels et al., 2007; Mayor et al., 2007; Kawano et al., 2010a; Keestra et al., 2013). *Drosophila* Toll, and the homologous Toll-like receptors in animals are PRRs that act as immune receptors of microbes. Both *Salmonella typhimurium* and *Shigella flexneri* are pathogens that invade host animal cells using a type III secretion system that is able to inject their effectors into host cells. *Salmonella* stimulates these responses by delivering through its type III secretion system the bacterial effector proteins SopE, SopE2, and SopB, which in a redundant fashion stimulate Rho family GTPases leading to the activation of MAPK and signaling by the transcription factor Nuclear factor-κB (NF-κB; Bruno et al., 2009). The NLR protein NOD1 senses cytosolic microbial products by monitoring the activation state of Rac/Rop family proteins including HsRac1, HsRhoA HsCdc42 (Keestra et al., 2013). Activation of HsRac1 and HsCdc42 by bacterial delivery or ectopic expression of SopE, an effector protein of *Salmonella*, triggered NOD1 signaling. In concert with a downstream kinase of NOD1, Receptor-interacting protein 2 (RIP2) mediated the induction of NF-κB-dependent inflammatory responses. Similarly, activation of the NOD1 signaling pathway by peptidoglycan (PGN) required HsRac1 activity. CA-HsRac1, HsCdc42, and HsRhoA activated the NOD1 signaling pathway.

Furthermore, GEF-H1 is a central component of pathogen recognition by NOD1 in animals (**Figure 4**; Fukazawa et al., 2008). Together, GEF-H1 and NOD1 not only detect the presence of PGN-derived muropeptides but also signal in response to *Shigella* effectors in the cytoplasm. GEF-H1 is recruited into bacterial invasion sites of *S. flexneri*, and subsequent *Mus musculus* RhoA (MmRhoA) small GTPase activation is required for cell invasion. In addition, GEF-H1 is requisite for the activation of NF-κB-dependent gene expression during *Shigella* invasion. GEF-H1 interacts with NOD1 and is required for NF-κB activation in response to PGN degradation products. Importantly, the *Shigella* effectors IpgB2 and OspB activate NF-κB by a mechanism that depends on both NOD1 and GEF-H1 and requires Rho-associated kinase (Rho-kinase) activation. GEF-H1 is a central component in a detection system that directs NF-κB activation in MmRhoA- and RIP2-dependent pathways initiated by the action of bacterial effectors and intracellular pathogen pattern recognition.

In animals, the host indirectly senses the pathogen by monitoring for cytotoxic necrotizing factor 1 (CNF1), an *Escherichia coli*-derived effector molecule (Boyer et al., 2011). CNF1 modifies *Drosophila melanogaster* Rac2 that then interacts with the innate immune adaptors Immune deficiency (IMD) and RIP1-RIP2 in fly and animal cells, respectively, to trigger an immune response.

Previous genetic studies have demonstrated that plant R protein functions are determined by multiple (co)chaperone proteins including SGT1, RAR1, and HSP90 (Shirasu, 2009). Interestingly, *Homo sapiens SGT1* RNAi prevents multiple cellular responses associated with NOD1 activation, indicating that HsSGT1 positively regulates NOD1 activation (da Silva Correia et al., 2007). Knockdown of *MmSGT1* or chemical inhibition of MmHSP90 abrogates inflammasome activity, and inhibition of MmHSP90 blocks NOD2-mediated activation of NF-kB and reduces NLR protein NALP3-mediated gout-like inflammation in mice (Mayor et al., 2007). The components of signal transduction in ETI are conserved among species. We noted that, in animals, NOD1 monitors the activation state of the Rho family proteins that are targeted by virulence effectors produced by pathogenic microbes (**Figure 4**). The mechanism for recognizing NLR proteins in animals shows striking similarities with the NLR protein recognition mechanism in plants through host proteins, called guardees, such as *Arabidopsis* RIN4 (Jones and Dangl, 2006). In contrast, OsRac1 appears to act downstream of the NLR proteins. At present, the precise differences in the roles of Rho family proteins in ETI signaling between animals and plants are largely unknown. Further studies are necessary to clarify these relationships.

## *IN VIVO* MONITORING OF Rac/Rop ACTIVATION

**FIGURE 6:**
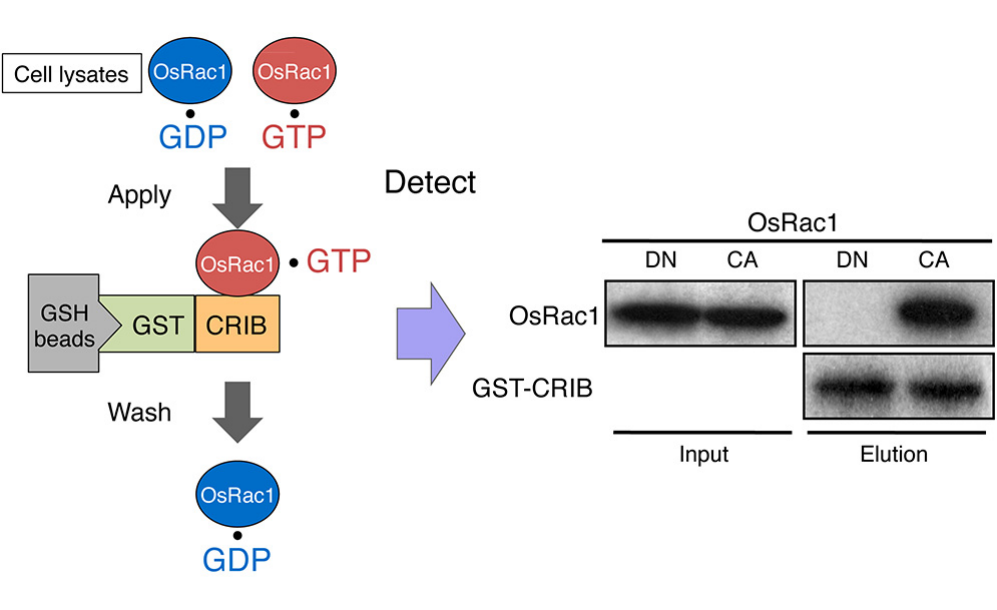
***In vivo* monitoring of Rac/Rop activation using a GST-CRIB pull-down assay.** The CRIB domain of PAK has a high affinity for the active GTP-bound form of Rac/Rop. PAK-CRIB-binding to Rac/Rop suppresses the intrinsic and catalytic rates of GTP hydrolysis of Rac/Rop that make it possible to purify the active constitutively active (CA) form but not the inactive dominant negative (DN) form of OsRac1 from cell lysates (Kawano et al., 2010a). A figure from Kawano et al. (2010a) was adapted for **Figure 6**.

**FIGURE 7:**
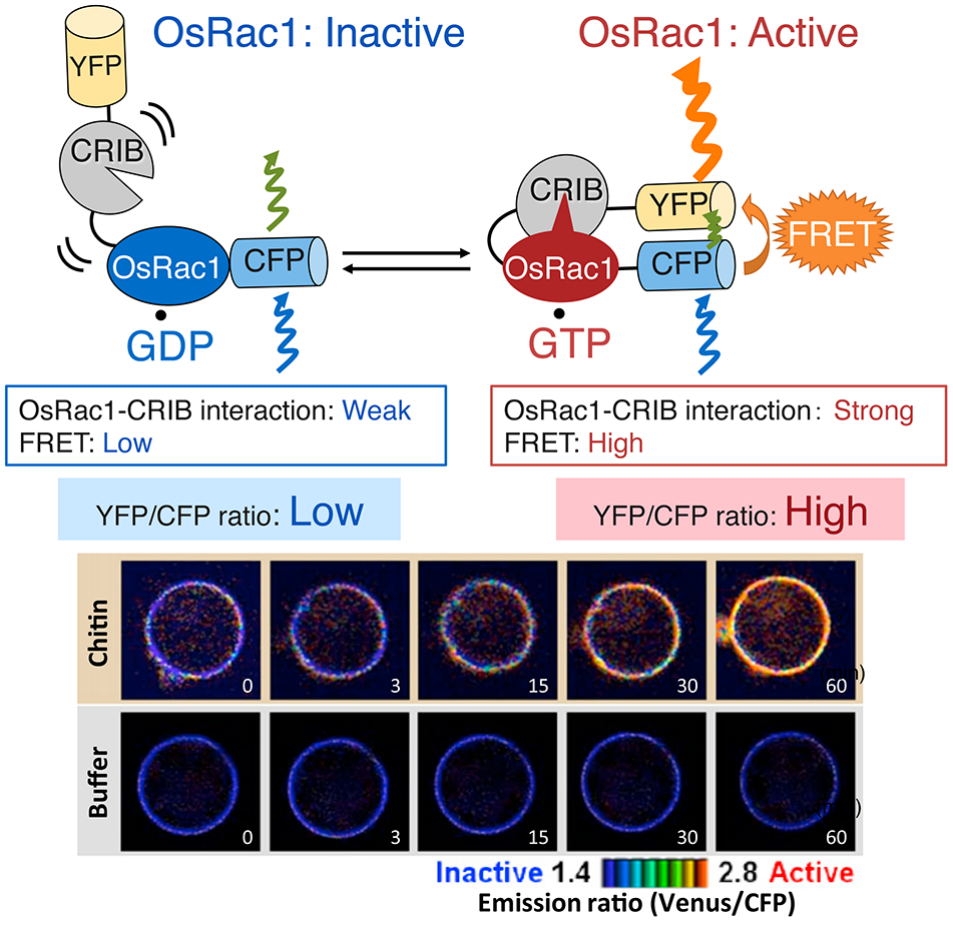
***In vivo* monitoring of Rac/Rop activation using the Raichu- Förster resonance energy transfer (FRET) sensor.** Intramolecular binding of active GTP.OsRac1 to CRIB moves CFP and YFP closer, thus enabling FRET (Kawano et al., 2010a). The resulting YFP fluorescence provides an estimate of the activation state of OsRac1 *in vivo*, with low and high ratios of YFP/CFP fluorescence corresponding to low and high levels of OsRac1 activation, respectively. OsRac1 is activated within 3 min after sensing chitin (Akamatsu et al., 2013). A figure from Akamatsu et al. (2013) was adapted for **Figure 7**.

Given that Rac/Rop is a master regulator controlling plant immunity, monitoring its activation within plant cells is believed to be the next key step in understanding plant immunity. Traditionally, small GTPase activities are measured using *in vivo* labeling of cells with of inorganic [^32^P] phosphate followed by isolation of the GTPase and thin-layer chromatography of bound guanine nucleotides. This method provides quantitative data for GDP and GTP levels on small GTPases but is a time-consuming procedure that requires large amounts of radioisotopes. Currently, we are able to use two alternative non-radioactive techniques, a PAK-CRIB pull-down assay and a Raichu-Förster resonance energy transfer (FRET) sensor, to monitor the *in vivo* activation of Rac/Rop (**Figures 6** and **7**; Sander et al., 1998; Mochizuki et al., 2001; Tao et al., 2002; Kawano et al., 2010a). These methods exploit the selective interaction of the CRIB of the Rac-effector PAK1 in animals. Since the CRIB-domain of PAK has a high affinity for the active GTP-bound form of Rac/Rop and PAK-CRIB binding results in a significantly reduced intrinsic GTPase activity of Rac/Rop, these factors result in an ideal tool for affinity purification of active GTP-bound forms of Rac/Rop from crude cell lysates. Recombinant GST-tagged PAK-CRIB protein is currently available from several manufacturers. GST-tagged PAK-CRIB allows one to “pull-down” the PAK-CRIB/GTP.Rac/Rop complex with glutathione affinity beads (**Figure 6**). Therefore, the assay provides a simple means of quantifying Rac/Rop activation in cells. The amount of activated Rac is determined by immunoblotting. This approach has greatly accelerated and, thus, simplified the semi-quantitative measurement of Rac activity in plants and animals (Sander et al., 1998; Tao et al., 2002; Xu et al., 2010).

We have established a FRET probe, Ras and interacting protein chimeric unit (Raichu)-OsRac1, for monitoring the activation of OsRac1 in living cells (**Figure 7**; Mochizuki et al., 2001; Kawano et al., 2010a). Raichu was originally developed to study the activation of various small GTPases, including Rac1, in mammalian cells (Mochizuki et al., 2001; Itoh et al., 2002). Raichu and its variants are well-established tools for monitoring the activation of small GTPases among species. Raichu-OsRac1 is composed of OsRac1, the CRIB domain of PAK1, which binds specifically to the GTP-bound form of OsRac1, and the FRET donor (YFP) and the FRET acceptor (CFP). Intramolecular binding of active GTP.OsRac1 to CRIB brings CFP closer to YFP, enabling FRET from CFP to YFP to occur. The resulting YFP fluorescence provides an estimate of the activation state of OsRac1 *in vivo*, with low and high ratios of YFP/CFP fluorescence corresponding to low and high levels of OsRac1 activation, respectively. Using Raichu-OsRac1, we identified the R protein Pit that activated OsRac1 on the plasma membrane (Kawano et al., 2010a) and observed that OsRac1 was activated within 3 min after sensing chitin, a cell wall component of the rice blast fungus (**Figure 7**; Akamatsu et al., 2013). The Raichu system is powerful tool for monitoring activation states of small GTPase, thus, we hope that this system becomes widely used in the field of plant biology for understanding the spatio-temporal characteristics of small GTPase activation.

## CONCLUDING REMARKS

The study of Rac/Rop family GTPase-dependent plant immunity is a rapidly expanding field. Recently, progress has been made in elucidating the defense mechanisms of rice OsRac1 to rice blast fungus (**Figures 3** and **4**) and barley HvRacB to powdery mildew (**Figure 5**). The detection of pathogen-derived effectors using NLR proteins between plants and animals is highly conserved (**Figure 4**). There is a high likelihood that the NLR proteins arose from a primitive innate immune system, and it will be interesting to elucidate the evolutionary process. Currently, our knowledge of the downstream signaling components in ETI is limited. More biochemical and structural studies are required to understand the possible mechanisms in ETI.

## Conflict of Interest Statement

The authors declare that the research was conducted in the absence of any commercial or financial relationships that could be construed as a potential conflict of interest.
